# Midlife vulnerability and food insecurity: Findings from low-income adults in the US National Health Interview Survey

**DOI:** 10.1371/journal.pone.0233029

**Published:** 2020-07-13

**Authors:** Lisa M. Soederberg Miller, Daniel J. Tancredi, Lucia L. Kaiser, Jeffrey T. Tseng

**Affiliations:** 1 Department of Human Ecology, University of California, Davis, CA, United States of America; 2 Center for Healthcare, Policy, and Research, UC Davis School of Medicine, Sacramento, CA, United States of America; 3 Nutrition Department, University of California, Davis, CA, United States of America; 4 Communication Department, University of California, Davis, CA, United States of America; Indiana University Purdue University at Indianapolis, UNITED STATES

## Abstract

**Background:**

Food insecurity, limited access to adequate food, in adulthood is associated with poor health outcomes that suggest a pattern of accelerated aging. However, little is known about factors that impact food insecurity in midlife which in turn could help to identify potential pathways of accelerated aging.

**Methods:**

Low-income adults (n = 17,866; 2014 National Health Interview Survey), ages 18 to 84, completed a 10-item food security module and answered questions regarding health challenges (chronic conditions and functional limitations) and financial worry. We used multinomial logistic regression for complex samples to assess the association of health challenges and financial worry with food insecurity status and determine whether these associations differed by age group, while adjusting for poverty, sex, race/ethnicity, education, family structure, social security, and food assistance.

**Results:**

Food insecurity rates were highest in late- (37.5%) and early- (36.0%) midlife, relative to younger (33.7%) and older (20.2%) age groups and, furthermore, age moderated the relationship between food insecurity and both risk factors (interaction p-values < .05, for both). The effects of poor health were stronger in midlife relative to younger and older ages. Unlike younger and older adults, however, adults in midlife showed high levels of food insecurity regardless of financial worry.

**Conclusions:**

Findings suggest that food insecurity in midlife may be more severe than previously thought. Greater efforts are needed to identify those at greatest risk and intervene early to slow premature aging.

## Introduction

Food insecurity, defined as the inability to afford and access nutritious foods to eat, disproportionately affects those living in poverty and leads to poor health, higher healthcare costs, and increased risk of mortality [1–7]. Individuals living with food insecurity are at increased risk of poor quality diet and inadequate nutrient intake, which contribute to muscle mass loss, mobility problems, and frailty at earlier ages than those living with adequate access to nutritious food [8–15]. Older adults in the US often have lower rates of food insecurity than do working age (e.g., 25–61 years of age) and young adults, possibly because of social safety nets in the US such as social security [13, 16–24]. It is unclear, however, how food insecurity rates in the middle portion of adulthood compare to earlier and later ages. Some studies have shown a steady decline in food insecurity across adulthood [22, 24] while others have shown stability [23], or a curvilinear relationship with a peak at age 45 in a sample of 18 to 64 year olds [25]. There is some indication that chronic conditions and disability may impact food insecurity to a greater extent in midlife relative to other age groups [20], factors which may contribute to the ambiguity in the literature. Midlife or “middle-age” represents the life course position between young adulthood and old age. Although lacking a clear beginning or end, this period is often defined as beginning at age 40 or 45 and ending at age 60 or 64 [26, 27]. As described below, middle-aged adults experience changes in social, psychological, and biological factors that are unique to this portion of the lifespan [27, 28] and could increase the risk of food insecurity in midlife. In the present study, we explored two potential moderators of the relationship between age and food insecurity: health challenges and financial worry.

### Health challenges

Midlife is typically when the onset of chronic disease and functional limitations occurs [27]. Health challenges are more likely, in terms of number and severity, among low-income, relative to high-income, middle-aged adults [29, 30]. Poor health in midlife may be connected to food insecurity through several paths including reduced ability (e.g., mobility, strength, dexterity) to locate, access, and prepare inexpensive nutritious food. We hypothesized that the changes associated with midlife are likely to exacerbate the impact of health challenges on food insecurity. Still, it is unclear whether the presence of health challenges or another factor, in particular, financial worry, best captures the moderating effects of age on food insecurity.

### Financial worry

Another hallmark of midlife is an increase in the number of social roles (e.g., related to work, parenting, and other forms of caregiving) [26]. In particular, middle-aged adults often care for children and aging parents simultaneously, which has earned current cohorts the name *sandwich generation*, or more recently, *pivot generation* [31, 32]. Among low-income, middle-aged adults, additional roles may contribute to greater financial worry, defined as concerns regarding one’s ability to meet basic financial needs and obligations, and maintain a standard of living. Research has shown that financial worry is closely connected to stress and psychological well-being [33, 34], which in turn increase vulnerability to food insecurity [35]. As with research on physical health, evidence suggests that poor psychological health increases the risk of poor quality diet and food insecurity [36, 37]. Yet, few if any studies have examined associations between financial worry and food insecurity. We hypothesized that financial worry would moderate the relationship between age and food insecurity due middle-aged adults’ concerns surrounding their caregiving responsibilities and self-care needs relative to their financial concerns. In midlife, financial worry may reflect perceptions surrounding increasing risk of lost work time, reduced prospect of re-employment [7, 38], and few safety-net opportunities (relative to parents of younger children or older adults) [20, 39]. Middle-aged adults may incur expenses associated with caring for adult children or aging parents that may not be factored into eligibility tests because care recipients reside outside, or transition in and out of, the household [40, 41]. Even when eligible, middle-aged adults may not be aware of benefits as evident in lower participation in food assistance programs, such as the Supplemental Nutrition Assistance Program (SNAP) [42].

**Fig 1:**
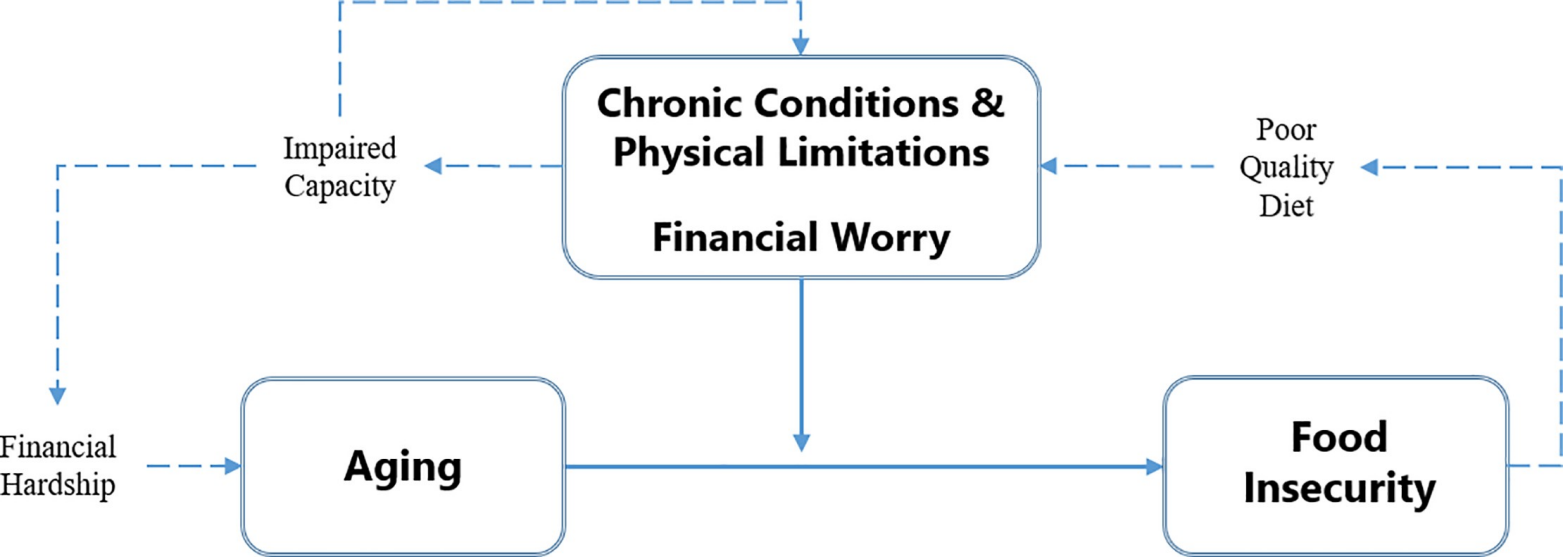
Conceptual model of accelerated aging and food insecurity in midlife: Cascading impact of poor diet quality, health challenges, financial hardship, and financial worry.

Thus, midlife represents the intersection of declining health, increasing financial uncertainty, and multiple roles which often include caregiving responsibilities. In the present study, we explored the possibility that health challenges and financial worry, independently moderate the relationship between age and food insecurity such that the relationships are most pronounced in midlife. The hypothesized relationships between health challenges, financial worry, and food insecurity are depicted in Fig 1 [6, 13, 35, 36, 43, 44].

## Methods

We analyzed cross-sectional data from the 2014 National Health Interview Survey (NHIS). The NHIS is a study conducted by the National Center for Health Statistics, Centers for Disease Control and Prevention designed to track health status and health care access since 1957 [45]. The 2014 NHIS contains a nationally representative sample (n = 34,798) of US noninstitutionalized adults ages 20 to 84. Data were collected using a face-to-face interview format. In this study, we examined a subset of adults who were characterized as low income (n = 17,866) using the poverty-to-income ratio (PIR), which is the ratio of income to the poverty threshold set by the US Census Bureau (adjusted for inflation and family size). The poverty threshold for a family of 4 in 2014 was $23,850. Thus, income of this amount for a family this size would have a PIR = 1 (i.e., 100% of federal poverty threshold). We defined low-income as those with a PIR < 3, indicating less than three times the federal income threshold. This definition of low-income offers a good chance of including those with marginal food insecurity [21, 46].

### Measures

#### Dependent variable

Food insecurity was assessed using the US Department of Agriculture’s 10-item, Adult Food Security Scale, which is a widely-used measure assessing the frequency with which adults experienced, for example, not having enough money to buy food or were unable to afford to eat balanced meals during the 30-day period prior to survey [25]. Responses, ranging from 0 to 10, were categorized into 4 levels: food secure (score of 0), marginal food secure (score of 1–2), low food secure (score of 3–5), and very low food secure (score of 6–10) [47].

#### Independent variables

Age, measured in years ranging from age 18 to 84, was stratified into four groups: young adults, ages 18–34; early midlife, ages 35–49; late midlife, ages 50–64; and older adults, ages 65–84. Because our focus was on midlife, we included an early- and late- midlife group. We defined the start of the early midlife group at age 35, based on recent evidence of increased health risks as young as 35 [48–50]. Age 50 marked the beginning of the late-midlife group, consistent with past work on midlife food insecurity [20]. The health challenges variable was constructed by crossing bivariate (yes/no) measures of chronic conditions with functional limitations. The chronic conditions variable was determined by scoring a “yes” if respondents had ever been told they had any of the following 13 chronic conditions, coronary heart disease, hypertension, angina pectoris, heart attack, heart condition/disease, hepatitis, cancer, stroke, COPD, asthma, kidney, diabetes, arthritis [51]. The functional limitations variable was similarly constructed based on a list of 12 activities. Respondents received a “yes” if they indicated difficulty performing any of the following activities without special equipment, walk 1/4 mile, climb 10 steps, stand 2 hours, sit 2 hours, stoop/bend or kneel, reach overhead, grasp small objects, lift/carry 10 lbs, push large objects, go out to events, participate in social activities, relax at home. The health challenges composite variable combined the two variables into 3 levels: 1 = neither a chronic condition nor a functional limitation; 2 = either one but not both; 3 = both (at least one chronic condition and at least one functional limitation) [23]. A financial worry was assessed using six questions pertaining to financial worry related to paying monthly bills, paying rent/ mortgage/ housing, and other costs. Responses were made on a scale of 1 (not worried at all) to 4 (very worried) and a composite score was formed by averaging across the 6 items.

#### Control variables

We controlled for poverty level, sex, race/ethnicity, education level, presence of older adults in the home (other than self), presence of children in the home, household social security benefits, and household SNAP benefits. Poverty PIR was stratified into three groups: extreme poor- less than the poverty line (<1 PIR); very poor (1 to <2 PIR); and poor (2 to <3 PIR). Sex was a dichotomously coded as male/female. Race/ethnicity was assessed using four non-overlapping categories: White, Black, Hispanic, and other race. Education was coded into three groups: high school or lower; some college; and college graduate or more. To address the potential for caregiving responsibilities in the home, two family characteristics were assessed. First, presence of older adults in the home (other than self) was dichotomized into no/yes; and, second, presence of children in the home was categorized into three levels: no children; 1 child; and 2 or more children. Household social security and SNAP benefits were both dichotomized into no/yes.

### Analytic strategy

Analyses were conducted by using survey data analysis procedures in SPSS software (version 24), using the survey weight and study design variables provided by the 2014 NHIS [52], yielding inferences that represent the US civilian, noninstitutionalized adult population and design-adjusted variance estimates (for hypothesis testing and confidence interval estimation). Data were coded as missing for “refused,” “not ascertained,” “unsure,” and “don’t know” responses. First, we examined food insecurity prevalence, broadly defined, in terms of food secure (score of 0) and food insecure (scores of 1–10) using logistic regressions for complex samples. In two models, we tested age effects before and after adjusting for health, financial worry, poverty, sex, race/ethnicity, education, elder and child presence, social security and SNAP support. Second, we used multinomial logistic regressions for complex samples to examine food security rates at marginal, low, and very low food security levels relative to food secure. Our dependent variable is ordered and could be analyzed by ordinal logistic regression under the strong assumption of proportional odds, but we opted to avoid this assumption and use multinomial logistic regression instead, given our goal to characterize the joint and separate effects of age and functional limitations on varying levels of food insecurity. To examine whether the effects for focal risk factors (i.e., health and financial worry) were modified by age, age-by-risk factor interaction terms were added to the model in separate analyses. Wald tests were used to examine main effects and interaction, with *p* < .05, to evaluate significance. Effect sizes from multinomial logistic regression models were reported as relative risk ratios, where the relative risk concerns the probability for each level of the dependent variable relative to the probability of the reference level.

### Ethics statement

NHIS is approved by the Research Ethics Review Board of the National Center for Health Statistics and the U.S. Office of Management and Budget. All NHIS respondents provided oral consent prior to participation. All data in the publicly available dataset are fully anonymized prior to release. All authors declare they have no competing interests.

## Results

Overall, 32.9% of low-income respondents (n = 17,866) reported being food insecure in the 30 days prior to the survey. Food insecurity rates increased from young, 33.7%, to early-mid, 36.0%, and late-mid, 37.5%, representing a 10% increase in food insecurity rates from young adulthood to late midlife (Table 1). As presented in the top-left portion of Table 2, late middle-aged adults showed an increased risk of food insecurity (RRR = 1.18; 95% CI = 1.05, 1.33) relative to young adults. Older adults had the lowest rates, 20.2%, representing a significantly decreased risk (RRR = 0.50; 95% CI = 0.44, 0.57) relative to young adults.

**Table 1:** Characteristics of low-income adults by food security status (National Health Interview Survey, 2014; n = 17,866).

			Food Security Status				
	Sample		Secure	Marginal	Low	Very Low	
Characteristic	n	Column %	Row %	Row %	Row %	Row %	*P* value^a^
Total	17866			12.1	11.3	9.5	
Age							<0.0001
Younger (18–34)	6026	0.383	0.663	0.135	0.114	0.088	
Early Middle (35–49)	4179	0.240	0.640	0.129	0.127	0.103	
Late Middle (50–64)	4011	0.214	0.625	0.119	0.126	0.130	
Older (65–84)	3650	0.162	0.798	0.075	0.076	0.051	
Poverty Level (PIR < 3)							<0.0001
Low (PIR < 1)	5360	0.300	0.531	0.154	0.162	0.153	
Mid (1> = PIR < 2)	7111	0.398	0.664	0.125	0.120	0.091	
High (2> = PIR < 3)	5396	0.302	0.820	0.082	0.056	0.042	
Health Challenges							<0.0001
Neither FL nor CC	7349	0.412	0.728	0.118	0.097	0.057	
One (either FL or CC)	5012	0.281	0.674	0.126	0.106	0.094	
Both (FL and CC)	5476	0.307	0.593	0.119	0.142	0.146	
Financial Worry (yes)	5401	0.309	0.465	0.155	0.189	0.191	<0.0001
Sex (female)	9612	0.538	0.656	0.124	0.120	0.100	<0.01
Race/Ethnicity							<0.0001
Non-Hispanic White	9558	0.535	0.717	0.103	0.089	0.091	
Non-Hispanic Black	2876	0.161	0.549	0.154	0.163	0.134	
Hispanic	4163	0.233	0.636	0.141	0.142	0.081	
Multirace/other	1251	0.070	0.713	0.115	0.096	0.076	
Education							
< = High School Diploma	7439	0.417	0.614	0.135	0.142	0.109	<0.0001
Some College	6993	0.392	0.669	0.124	0.105	0.102	
> = College Degree	3390	0.190	0.798	0.083	0.068	0.051	
Older Adults (yes)	2305	0.129	0.783	0.088	0.078	0.051	<0.0001
Children							<0.0001
None	10201	0.571	0.692	0.098	0.107	0.103	
One	2805	0.157	0.651	0.146	0.121	0.082	
Two or more	4860	0.272	0.638	0.153	0.123	0.086	
Social Security (yes)	5319	0.298	0.691	0.100	0.109	0.100	<0.0001
SNAP Benefits (yes)	5159	0.289	0.471	0.170	0.186	0.173	<0.0001

^a^ based on Chi-Square tests

^FL = Functional Limitation; CC = Chronic Condition; SNAP = Supplemental Nutrition Assistance Program^

**Table 2:** Effect size estimates of food security among young, early mid-, late mid-, and older adults, unadjusted and adjusted for demographic characteristics, health challenges, and financial worry (National Health Interview Survey, 2014; n = 17,866).

Age	Food Security Status	Unadjusted Effect Size Estimates			Adjusted Effect Size Estimates		
		Preliminary Models					
Young vs:	Secure vs:	RRR	95% CI		RRR	95% CI	
Early Middle (35–49)	Insecure	1.11	0.99	- 1.24	0.81	0.71	- 0.92
Late Middle (50–64)	Insecure	1.18	1.05	- 1.33	0.69	0.59	- 0.80
Older (65–84)	Insecure	0.50	0.44	- 0.57	0.37	0.30	- 0.46
		Model 1			Model 2		
Young vs:	Secure vs	RRR	95% CI		RRR	95% CI	
Early Middle (35–49)	Marginal	0.99	0.84	- 1.17	0.80	0.67	- 0.97
	Low	1.16	0.98	- 1.37	0.84	0.70	- 1.01
	Very Low	1.22	1.00	- 1.47	0.75	0.61	- 0.93
Late Middle (50–64)	Marginal	0.93	0.78	- 1.11	0.76	0.61	- 0.94
	Low	1.17	0.98	- 1.39	0.68	0.55	- 0.84
	Very Low	1.57	1.30	- 1.91	0.59	0.47	- 0.75
Older (65–84)	Marginal	0.46	0.38	- 0.56	0.47	0.35	- 0.63
	Low	0.55	0.45	- 0.68	0.40	0.30	- 0.53
	Very Low	0.48	0.37	- 0.63	0.25	0.18	- 0.35

**Fig 2:**
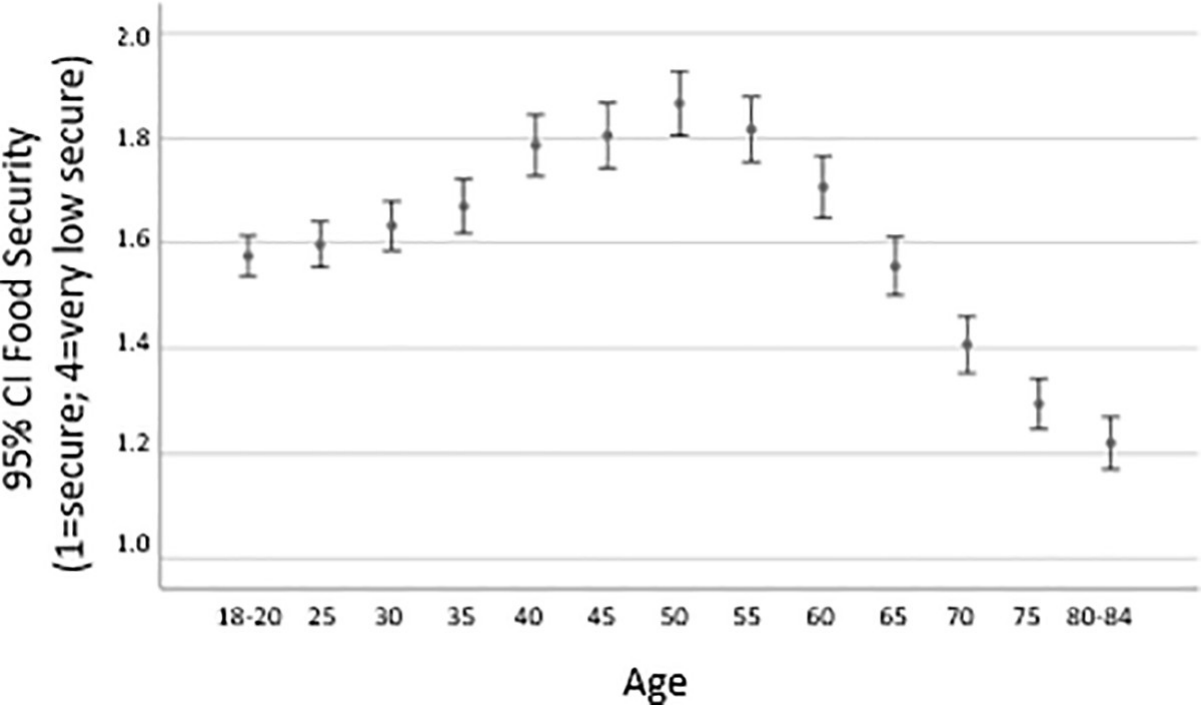
Food security means (unadjusted) by age (3- to 5-year increments).

We examined food insecurity in greater detail using four categories (secure, marginally secure, low secure, very low secure). Food insecurity rates decreased with increasing severity, 12.1%, 11.3%, 9.5%, for marginal, low, and very low food security levels, respectively (Table 1). We tested age differences and age moderation using multinomial logistic regression models, with food insecurity level as the dependent variable (with *food secure* as the reference level) and age group (with young adults as the reference group) as the independent variable. Fig 2 displays unadjusted food security means (i.e., ranging from 1 (food secure) to 4 (food very low secure) in small age increments (3–5 years) to illustrate the gradual change across the young, early-mid, late-mid, and older age groups.

In Model 1, the unadjusted relative risk ratios for marginal food security were nonsignificant among early- (RRR = 0.99; 95% CI = 0.83, 1.17) and late- (RRR = 0.93; 95% CI = 0.78, 1.11) middle-aged adults, indicating the relative risk of marginal food security (versus food security) for the midlife groups did not differ statistically from those of young adults (see bottom left portion of Table 2). On the other hand, the relative risk of very low food security was greater for both midlife groups (early-middle: RRR = 1.22; 95% CI = 1.00, 1.47; late-middle: RRR = 1.57; 95% CI = 1.30, 1.91) relative to younger adults. Very low food security increased 32.5% from young to late midlife (8.8% vs. 13.0%, see Table 1). Moreover, the late-midlife group was the only age group that did not decline from low- to very-low food security status (12.6% vs. 13.0%). Older adults, on the other hand, were less likely to be food insecure than young adults at all three levels of food security (marginal: RRR = 0.46; 95% CI = 0.38, 0.56; low: RRR = 0.55; 95% CI = 0.45, 0.68; very low: RRR = 0.48; 95% CI = 0.37, 0.63).

The likelihood of food insecurity changed, however, after adjusting for demographic characteristics and moderators (health challenges and financial worry) in Model 2. As shown in Table 2, the relative risk of food insecurity significantly declined (RRRs ranged from 0.25 to 0.80) in early-middle, late-middle, and older adulthood, relative to young adulthood (except for risk of low food security for early-middle aged adult, RRR = 0.84; 95% CI = 0.70, 1.01). Model 2 also showed that, after adjusting for demographic variables, health challenges, Wald F = 140.92, and financial worry, Wald F = 526.16, were significantly associated with food insecurity (Tables 2 and 3).

**Table 3:** Test statistic for age (Model 1), main effects (Model 2), and main effects plus moderation (Models 3 and 4) models (National health interview survey, 2014; n = 17,866).

Model	Source	df		Wald F/Chi-Square	*P*-value
Model 1	Age	9		191.63	.000
Model 2	Age	9		95.86	.000
	Poverty Level (PIR < 3)	6		116.88	.000
	Health Challenges	6		140.92	.000
	Financial Worry	3		526.16	.000
	Sex	3		0.41	.938
	Race/Ethnicity	9		56.02	.000
	Education	6		37.72	.000
	Older Adults in HH	3		21.53	.000
	Children in HH	6		41.06	.000
	Social Security Benefits	3		8.17	.043
	SNAP Benefits	3		152.91	.000
Model 3	Age x Health Challenges	18	17401	1.91	.012
Model 4	Age x Financial Worry	9		39.21	.000

*Note*: PIR = Poverty Income Ratio; HH = Household; SNAP = Supplemental Nutrition Assistance Program

**Fig 3:**
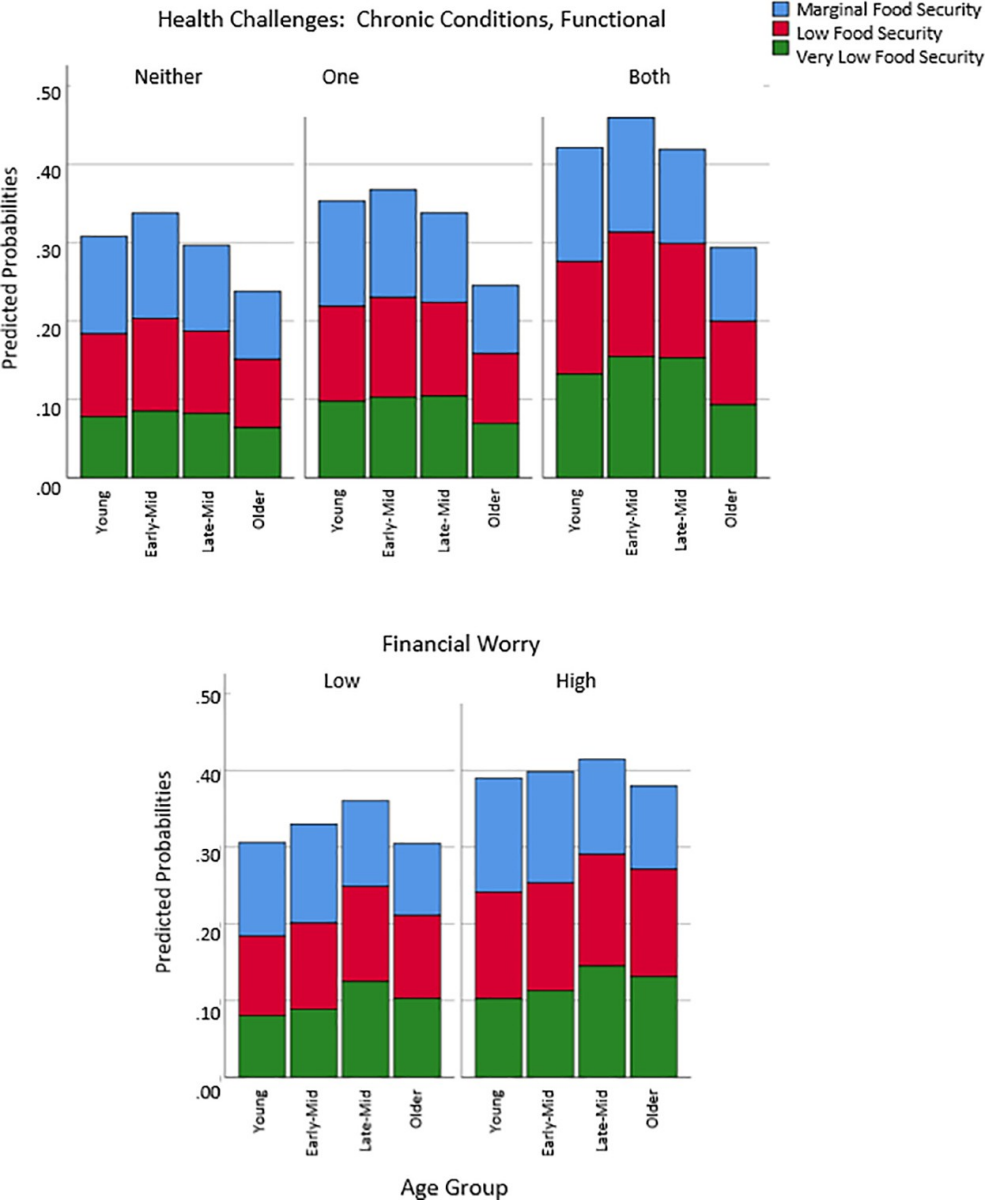
Predicted probabilities of food security (marginal, low, and very low) for age and health challenges (top) and age and financial worry (bottom) moderation models, by young adult (18–34 years), early midlife (35–49 years), late midlife (50–64 years) and older adults (65–84 years).

We tested age moderation of health challenges (Model 3) and financial worry (Model 4) on food insecurity after adjusting for demographic and moderator main effects (Model 2). Fig 3 shows predicted probabilities of marginal, low, and very low food security from Model 3 (top) and Model 4 (bottom). The Age x Health Challenges interaction term in Model 3 was significant, Wald F = 1.91 (Table 2). The top portion of Fig 3 indicates that the two middle-aged groups had the greatest risk of low and very low food security at the highest level of health challenges. When health challenges were lowest, the risk was comparable for young and late middle (low) and for young, early- and late- middle (very low food security). Marginal food security was largely unaffected by health.

In Model 4, the Age x Financial Worry interaction term was also significant, Wald F = 39.21 (Table 3). As shown in bottom portion of Fig 3, predicted probabilities generally increased from low- to high- financial worry; however, increases in marginal food security rates were small for all groups but young adults. In addition, the effects of financial worry were greatest for very low food security rates for young, early-middle, and older adults; however, for late-middle aged adults, very low and low food security rates were comparable. Thus, the hypothesis that middle-age adults would show greater effects of financial worry than younger or older adults was not supported.

## Discussion

Overall food insecurity rates were highest for late- (37.5%) and early- (36.0%) middle-aged adults, followed by young (33.7%) and older (20.2%) adults. In addition, relative to younger adults, middle-aged adults had an increased risk of low and very low food security when health challenges were greatest and older adults had a decreased risk of marginal, low, and very low food security, across all three levels of health challenge. These findings are consistent with research showing that food insecurity is associated with functional limitations and poor health [6, 7, 21, 35, 53, 54] as well as with research showing that the impact of disability on food insecurity is stronger for working-age adults (ages 25–61) than it is for younger and older adults [16]. The findings add to past work by specifying that period in which disability and health challenges may pose the greatest vulnerable; specifically later, rather than earlier, within the working-age range.

We also found that financial worry was positively associated with food insecurity. Past studies have included measures of financial strain or worry that clearly overlapped with food insecurity measures (having enough money to pay for clothing or food) [55], making it difficult to distinguish between financial worry and food insecurity. Our findings indicate that concerns surrounding one’s economic situation predict food insecurity even after adjusting for covariates and, furthermore, that the relationship between financial worry and food insecurity risk is moderated by age. Contrary to our expectations, however, food insecurity rates of younger and older adults, but not middle-aged adults, increased from low to high levels of financial worry. Food insecurity rates were high among middle-aged adults at both levels of financial worry.

When examined together, findings from the present study suggest that the negative effects of health challenges (chronic illness and functional limitations) on food insecurity are most pronounced among middle-aged adults nearing old age relative to other periods of adulthood. The findings are consistent with a pattern of midlife vulnerability found in studies examining stress, disease prevalence, and mortality rates [56–58]. There are several possible reasons why adults in late-midlife may be particularly vulnerable to food insecurity, especially in the presence of health challenges. Poor health may restrict employment which reduces the financial resources for food as well as the potential for engagement in the social world, which further decreases health [59]. Moreover, with few social welfare options, the need to remain in the work force places additional strain on financial well-being and, ultimately, increases the risk and severity of food insecurity [60, 61]. On the other hand, social safety nets may improve access to food in later life [20]. Another, less optimistic, reason why vulnerability may be less evident in later life is that life expectancy is markedly lower among those who experience chronic economic, health, and food insecurity burdens [56, 57, 62]. Food insecurity has been linked to nutritional frailty, characterized by sudden loss of weight, strength, and muscle mass, which is a major risk factor for poor health (e.g., dementia, sarcopenia) [63, 64].

Although this study has several strengths including use of a nationally representative sample of adults, examination of midlife relative to both younger and older ages, and consideration of financial worry, it also has limitations. In particular, the findings are based on a cross-sectional survey with assessments of health challenges, financial worry, and food insecurity at only one point in time, precluding causal inferences. The food insecurity instrument, although widely used and validated in US populations, measures the respondent’s perception of the adequacy of household food supplies but does not assess local food availability (e.g., presence of or distance to supermarkets) or “socially acceptable” availability [47].

### Conclusions

The present study contributes to the literature by showing that midlife appears to be a period of increased vulnerability to food insecurity, setting the stage for premature aging. Additional research is needed to further specify and test the mechanisms underlying food insecurity [44] and provide an empirical base for the hypothesized relationships in Fig 1 [6, 7, 13, 35, 36, 43, 44]. Such research would inform a growing literature on the cumulative effects of hardships across the life course [62, 65, 66] and their role in accelerating aging [8, 64, 67–71]. Further exploration of midlife vulnerability is needed to identify prevention and screening (e.g., primary care) strategies designed to halt or slow disease progression, promote rehabilitation, and increase wellness into later life [72, 73].
